# Remaining Useful Life Prediction Using Dual-Channel LSTM with Time Feature and Its Difference

**DOI:** 10.3390/e24121818

**Published:** 2022-12-13

**Authors:** Cheng Peng, Jiaqi Wu, Qilong Wang, Weihua Gui, Zhaohui Tang

**Affiliations:** 1 School of Computer, Hunan University of Technology, Zhuzhou 412007, China; 2 School of Automation, Central South University, Changsha 410083, China

**Keywords:** dual-channel LSTM, feature difference, momentum smoothing, RUL

## Abstract

At present, the research on the prediction of the remaining useful life (RUL) of machinery mainly focuses on multi-sensor feature extraction and then uses the features to predict RUL. In complex operations and multiple abnormal environments, the impact of noise may result in increased model complexity and decreased accuracy of RUL predictions. At the same time, how to use the sensor characteristics of time is also a problem. To overcome these issues, this paper proposes a dual-channel long short-term memory (LSTM) neural network model. Compared with the existing methods, the advantage of this method is to adaptively select the time feature and then perform first-order processing on the time feature value and use LSTM to extract the time feature and first-order time feature information. As the RUL curve predicted by the neural network is zigzag, we creatively designed a momentum-smoothing module to smooth the predicted RUL curve and improve the prediction accuracy. Experimental verification on the commercial modular aerospace propulsion system simulation (C-MAPSS) dataset proves the effectiveness and stability of the proposed method.

## 1. Introduction

Since entering the age of industrialization, machinery and equipment are seen everywhere. However, due to the harsh environment and the impact of improper human operation, mechanical failure difficulties have also emerged one after another. It can be seen that mechanical failure has become a threat hindering social development. Ordinary people may be affected by mechanical failure at any time. Therefore, how to accurately evaluate the RUL of a machine before a failure occurs is of great significance.

The current prediction methods for RUL can be divided into four categories: physical models, statistical models, artificial intelligence models, and hybrid methods [1].

Technology based on physical model describes the degradation process of machinery by establishing a mathematical model through failure mechanisms or damage first principles [2]. Chan and Enright et al. [3] proposed a time-dependent physical crack propagation method for predicting the RUL of the turbo propulsion system. Using the enhanced risk analysis tool and material constants calibrated to IN 718 data, the effect of time-dependent crack growth on the risk of fracture in a turbo engine component demonstrated a generic rotor design and a realistic mission profile. El-Tawil et al. [4] introduced an analytic prognostic methodology based on nonlinear damage laws. It enables the assurance of high availability and productivity with less cost for industrial systems. Kacprzynski et al. [4] developed a gear health prediction model using the physical method of failure. The approach, based on a statistical model, estimates the RUL of the machinery by establishing a statistical model based on empirical knowledge [5]. Barraza-Barraza and Tercero-Gómez et al. [6] put forward a way to use exogenous variables to construct three autoregressive (AR) models to predict the RUL of aluminum plates. Three autoregressive models with exogenous variables (ARX) were constructed, and their capability to estimate the remaining useful life (RUL) of a process was evaluated following the case of the aluminum crack growth problem. An existing stochastic model of aluminum crack growth was implemented and used to assess RUL estimation performance of the proposed ARX models through extensive Monte Carlo simulations.

Physical model and statistical model methods generally require expert knowledge, which is more difficult for non-professionals to understand and implement, so they are limited in practical application. With the advancement of graphics processor technology and the improvements in computing power, artificial intelligence methods have developed. Artificial intelligence builds models by collecting data, which can reduce the difficulty of understanding professional knowledge. It is easy to apply to various industries [7]. Degradation monitoring has been carried out on life time accelerating data. Zhou et al. [8] used an LSTM network for defect prediction. Zhou et al. [9] proposed a new improved multi-scale edge-labeling graph neural network (MEGNN) to estimate the tool wear condition through the updated edge labels using a weighted voting method. Wen et al. [10] adopted a new residual convolutional neural network (ResCNN) to solve the problem of gradient disappearance and predict the RUL of turbofan engines. ResCNN applies the residual block that skips several blocks of convolutional layers by using shortcut connections, which can help to overcome vanishing/exploding gradient problems. Li et al. [11] proposed a domain-adaptive RUL prediction model by integrating adaptive batch normalization (AdaBN) into a deep convolutional neural network (DCNN). The improved AdaBN-DCNN model can not only improve the accuracy of the prediction but also adapt to the prognostic tasks under different DDs. The sliding time window (TW) and the improved piecewise linear RUL function are also used in this paper to improve the prediction capability of the model. Han Li et al. [12] and others designed a multi-scale convolutional neural network (MSCNN) to directly establish the relationship between monitoring data and RUL. The MS-DCNN has three multi-scale blocks (MS-BLOCKs) [13], where three different sizes of convolution operations are put on each block in parallel. This structure improves the network’s ability to learn complex features by extracting features of different scales. Wu et al. [14] applied an RUL prediction method based on a deep long short-term memory (DLSTM) network using multiple sensor time-series signals. Zhao et al. [15] designed a mode based long multi-head attention and LSTM. The model can select the key features in the time-series data, then input them into the LSTM layer to mine the internal connections, and finally obtain RUL predicted results through two fully connected layers. Aiming at the characteristics of high dimensionality, high lag, and complexity of engine data, a multi-scale attention-based bidirectional long short-term memory neural network model based on self-training weights was proposed by Qiu et al. [16]. Multi-scale features were extracted through bidirectional long short-term memory neural networks (BiLSTM) of different scales. To improve the accuracy of prediction, a fusion algorithm based on self-training weights was proposed, and an attention mechanism was introduced to screen features at different scales.

The hybrid method combines the two or more methods mentioned above to neutralize their characteristics. Zemouri et al. [17] used regression models and artificial neural networks to propose a hybrid method. Wang et al. [18] combined the similarity method and sparse learning to predict health indicators. Zhang et al. [19] constructed an aging model of the battery by developing a fusion technique consisting of a relevance vector machine and particle filter (PF) for RUL estimation. Yan et al. [20] presented a neoteric approach of RUL estimation of bearings, which can evaluate the degradation stage of bearings and exploit the optimal RUL prediction by a hybrid degradation tracing model. Wang et al. [21] proposed a hybrid prognostics method for the RUL prediction of rolling element bearings through using relevance vector machine regressions and exponential degradation models. Gou et al. [22] introduced a new hybrid ensemble data-driven method to accurately estimate the state of health and RUL of Li-ion batteries. Li et al. [23] presented a novel hybrid Elman-LSTM method for battery RUL estimation.

The neural network method has achieved good results, but current research in this area still has following problems:

(1) The influence of time characteristics on the life prediction of machinery may change in different operating environments. Given this phenomenon, how we choose useful time features to avoid the appearance of feature redundancy or invalid features remains a problem to be solved.
(2) For time characteristics, researchers frequently make the model by focusing on the size of the time features at a certain moment and ignoring the difference between the time features of two different moments. In fact, the change speed of the characteristics reflects the internal state of the machine and also reflects the health state of the machine.
(3) Generally, the RUL of the machine is smooth and stable; that is, the remaining life of the machine in a certain period should be similar and the number of fluctuations relatively rare. However, in harsh environments, the data sent back to the system by the sensor are commonly unclean. The neural network method learns and predicts based on data, so the RUL predicted by the neural network fluctuates up and down. The RUL curve is jagged and causes a large deviation from the ground RUL.

To solve those problems, this paper proposes a dual-channel LSTM method. The proposed method constructs the direct relationship between the raw data and the ground RUL without using any prior information and improves the learning ability of neural networks by using time characteristic values and characteristic differences to predict the RUL. First of all, some features can hardly change during the entire time period, and the amount of information carried is also very small. If all the features are directly input into the neural network, the training time is longer. Therefore, we adopt a method of adaptively selecting features to eliminate features that have not changed in the life cycle and solve the problem of data redundancy. Secondly, we extract the first-order difference results of features as the input of the model to reflect the change speed of features and improve the accuracy of RUL prediction. Thirdly, in this neural network, LSTM is used to calculate the spatial dimension relationship of different features at each time, and a convolutional neural network (CNN) is used to consider the relationship in the time dimension and fuse the features of multiple time moments into one dimension. Aiming at the jagged phenomenon of the RUL curve predicted by the neural network, a momentum-smoothing method is proposed to deal with RUL curves and improve the accuracy of prediction.

The rest of the paper is organized as follows. The Section 2 introduces the structure of the dual-channel LSTM. Section 3 discusses the degradation data of aircraft turbofan engines and shows the comparative experiments of the dual-channel LSTM network with the other latest methods and so on. The Section 4 gives conclusions and further work.

## 2. Methodology

**Figure 1:**
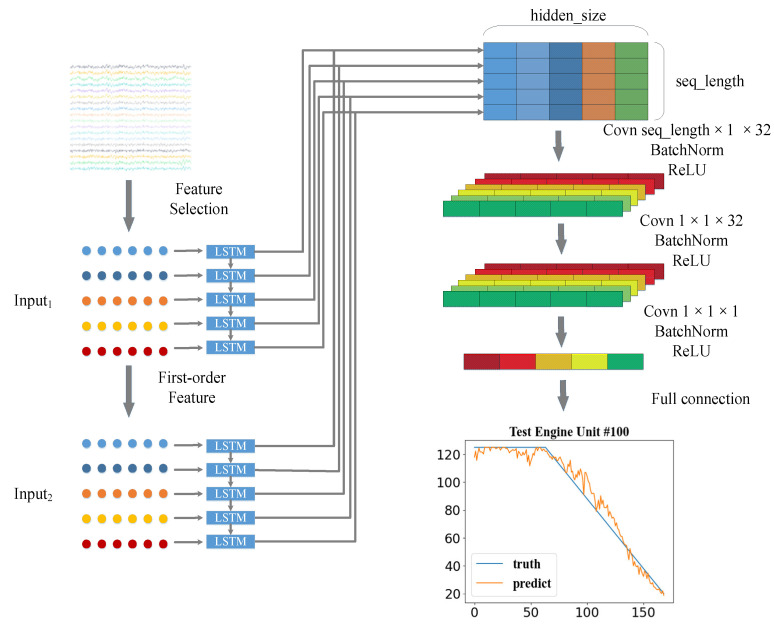
Dual-channel LSTM network architecture.

In this part, a new prediction structure for RUL estimation is proposed, called dual-channel LSTM. The architecture of the dual-channel LSTM network is presented in Figure 1. The whole algorithm can be divided into four parts: data preprocessing, dual-channel LSTM, RUL prediction, and momentum smoothing.

### 2.1. Data Preprocessing

The sample data we employed are C-MAPSS datasets from NSNA. The C-MAPSS dataset is composed of four diverse sub-datasets FD001, FD002, FD003, and FD004, which have the following related advantages and disadvantages. First, the sample size is sufficient. Each subsample contains a training set and test set, and the number of samples is acceptable. Second, there is a wide variety of samples. The whole training sample contains four sub-datasets. The data distribution of each sub-dataset is distinct, which is suitable for testing the generalization of the model. However, the sample is the data generated by simulating the real environment, and there is a certain deviation from the real sample of a turbine engine. At the same time, the sub-dataset contains a lot of noise, which is not conducive to the feature generalization of the model. Therefore, we must firstly preprocess the data, select features useful for model prediction, standardize the data, and speed up the convergence of the model. The following are segmented into the three aspects of feature selection, standardized processing, and time-window processing.

#### 2.1.1. Feature Selection

The following uses engine unit No. 1 in the FD001 sub-dataset as an example to visualize its operational settings and some sensor measurements.

**Figure 2:**
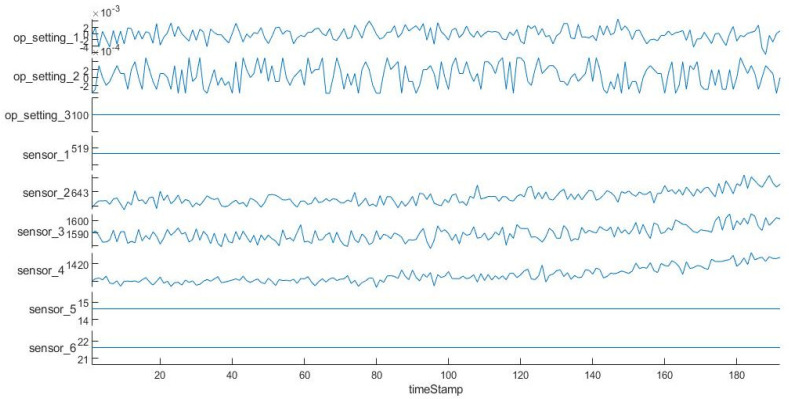
Partial time features of engine unit 1.

In Figure 2, the abscissa is time; the ordinate includes the operating settings of the engine and some sensors. In Figure 2 above, we can see that some time characteristics remain unchanged throughout the entire period, which has no positive effect on the prediction of RUL. It is necessary to choose the features that have a positive influence on the model prediction. Therefore, we can use prognosability to measure the variability of state indicators, and select time features that have changed significantly. The prognosability formula is as follows:

(1) $${\mathrm{prognosability}}_{j}=e^{\left(-\frac{std(x_{j})}{mean\left|x_{j}(1)-x_{j}\right|}\right)},j=1,\ldots ,M$$

where *x_j_* represents the measurement vector of a certain feature on the *j*-th system, the variable *M* is the number of systems to be monitored, and *x_j_* is the measurement vector at the first moment of the *j*-th system. The values of prognosability range from 0 to 1. For all features, if their prognosability is equal to 0 or NaN, these features are removed. Because these features have not changed in the whole prediction cycle, the results of each sub dataset selection are as follows:

FD001: op_setting_1, op_setting_2, sensor_2, sensor_3, sensor_4, sensor_6, sensor_7, sensor_8, sensor_9, sensor_11, sensor_12, sensor_13, sensor_14, sensor_15, sensor_17, sensor_20, sensor_21.

FD002: op_setting_1, op_setting_2, op_setting_3, sensor_1, sensor_2, sensor_3, sensor_4, sensor_5, sensor_6, sensor_7, sensor_8, sensor_9, sensor_10, sensor_11, sensor_12, sensor_13, sensor_14, sensor_15, sensor_16, sensor_17, sensor_18, sensor_19, sensor_20, sensor_21.

FD003: op_setting_1, op_setting_2, sensor_2, sensor_3, sensor_4, sensor_6, sensor_7, sensor_8, sensor_9, sensor_10, sensor_11, sensor_12, sensor_13, sensor_14, sensor_15, sensor_17, sensor_20, sensor_21.

FD004: op_setting_1, op_setting_2, op_setting_3, sensor_1, sensor_2, sensor_3, sensor_4, sensor_5, sensor_6, sensor_7, sensor_8, sensor_9, sensor_10, sensor_11, sensor_12, sensor_13, sensor_14, sensor_15, sensor_16, sensor_17, sensor_18, sensor_19, sensor_20, sensor_21.

#### 2.1.2. Normalization

The standardization of data is to scale the data to a small specific interval, which can speed up the model convergence. The data standardization method used in this article is the z-score. The formula is as follows:

(2) $$z=\frac{x-u}{\sigma }$$

where *u* represents the mean value of all selected features; σ represents the standard deviation of all selected features; and *x* represents the value of a chosen feature.

**Figure 3:**
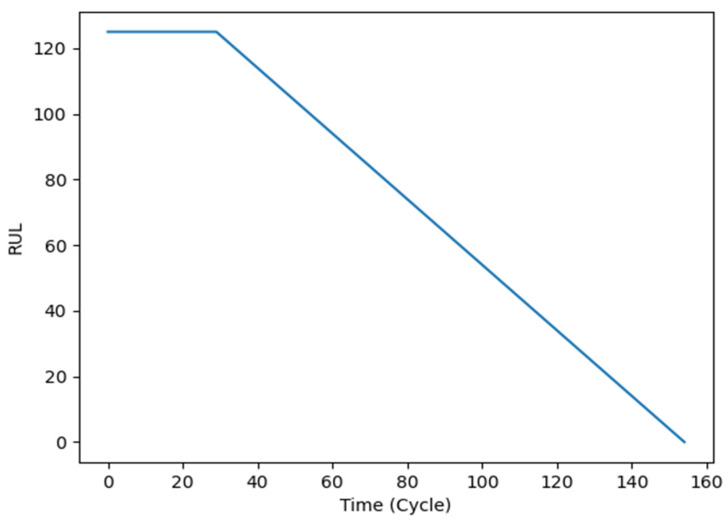
The curve of the ground RUL.

Considering that the mechanical state can be divided into a health state and a degraded state, the real RUL should be a piecewise linear function [24]. As shown in Figure 3, the ground RUL can be segmented into two parts: a constant portion and a linear degraded portion. Therefore, an RUL threshold needs to be set.

#### 2.1.3. Time-Window Processing

**Figure 4:**
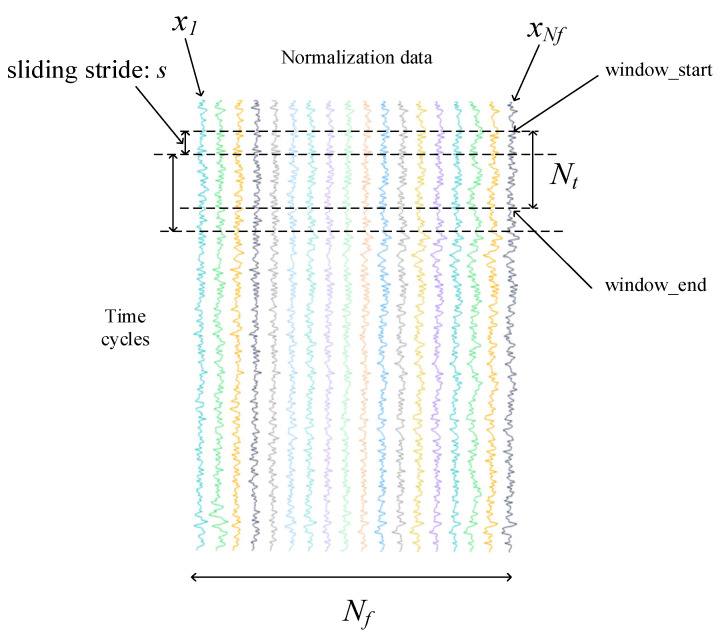
Time window.

The change speed of the characteristics reflects the internal state of the machine and the health state of the machine. Therefore, we can use the first-order difference results of features as the change speed of features to improve the accuracy of RUL prediction. Of course, adding higher-order difference results can also be considered to predict RUL, but the parameters of the model become more numerous. Then, there remain only features and feature differences to be considered. Two time windows need to be divided for network input. The specific operations are as follows:

First, divide the first time window. As shown in Figure 4, the window width is denoted as N_f_; the length of window is N_t_; the sliding stride is denoted as s (s = 1); and the feature value window Input_1_ = [*x*_1_, *x*_2_, …, *x_Nf_*].

(3) $$x_{i}=\left[\begin{array}{c} x_{i}^{window\_start}\\ x_{i}^{window\_start+1}\\ \vdots \\ x_{i}^{window\_end} \end{array}\right]\;i=1,\cdots ,N_{f}$$

The input size of Input1 is *N_t_* × *N_f_*. *N_f_* represents the number of features chosen by the prognosability formula. After the first time window is obtained, the time feature difference value can be obtained. The feature difference value window Input_2_ = [*d*_1_, *d*_2_, …, *d_Nf_*].

(4) $$d_{i}=\left[\begin{array}{c} x_{i}^{window\_start+1}-x_{i}^{window\_start}\\ x_{i}^{window\_start+2}-x_{i}^{window\_start+1}\\ \vdots \\ x_{i}^{window\_end}-x_{i}^{window\_end-1} \end{array}\right]\;i=1,\cdots N_{f}$$

The input size of Input2 is (*N_t_* − 1) × *N_f_*, and each pair of inputs has its corresponding RUL value, which is used for supervised training.

### 2.2. Dual-Channel LSTM Predicts Lifetime

When we use recurrent neural networks, in most cases, we deal with features at different times, ignoring another dimension, the feature difference. The feature difference at unequal times can let the network know the change rate of the feature value, assist the method to consider the feature information more comprehensively, reduce the influence of noise on the model, make the model more robust, and enhance the differentiation ability. Therefore, we can use dual-channel LSTM to process the eigenvalues at different times and the feature difference separately and calculate the spatial dimension relationship of different features at each time. This is described in detail below.

As shown in Figure 1 above, after the time window is processed, we can get two inputs, Input_1_ and Input_2_. Input_1_ represents the value of each time feature within a window time; Input_2_ represents the difference value of the time feature within the same window time. Then we use two LSTM networks to process Input_1_ and Input_2_. The output after the first recurrent neural network processes Input_1_ is Output_1_; Output_1_ = [*h*_1_, *h*_2_, …, *h_hidden_size_*].

(5) $$h_{i}=\left[\begin{array}{c} h_{i}^{window\_start}\\ h_{i}^{window\_start+1}\\ \vdots \\ h_{i}^{window\_end} \end{array}\right]\;i=1,\cdots ,hidden\_size$$

The output after the second recurrent neural network processes Input_2_ is Output_2_; Output_2_ = [*g*_1_, *g*_2_, …, *g_hidden_size_*].

(6) $$g_{i}=\left[\begin{array}{c} g_{i}^{window\_start+1}\\ \vdots \\ g_{i}^{window\_end} \end{array}\right]\;\;i=1,\cdots ,hidden\_size$$

The first line feature of Output_1_ can be obtained by the second line feature of Output_1_ and the first line feature information of Output_2_, so the first line feature of Output_1_ is not considered. Finally, we add the last (*N_t_*_−1_) row vectors of Output_1_ and Output_2_ directly to get Output; Output = [*o*_1_, *o*_2_, …, *o_hidden_size_*].

(7) $$o_{i}=\left[\begin{array}{c} h_{i}^{window\_start+1}+g_{i}^{window\_start+1}\\ \vdots \\ h_{i}^{window\_end}+g_{i}^{window\_start+1} \end{array}\right]i=1,\cdots ,hidden\_size$$

### 2.3. Life Prediction

RUL prediction can be divided into two parts. One part is to use CNN to consider the relationship in the time dimension and fuse the features of multiple time moments into one dimension after dual-channel LSTM processing, and another part is to use a fully connected neural network to predict RUL. The convolution part mainly includes convolution operation, batch normalization [25,26], and activation function. The main features of the convolutional layer are sparse interaction, parameter sharing, and equivariant representation. Among them, sparse interaction can play a regularization [27] role; parameter sharing reduces the number of parameters of the model and significantly increases the size of the network without increasing the training data.

As shown in Figure 1, the filter sizes of the three convolutional layers are selected as 32 × 1, 32 × 1, and 1 × 1. Each convolutional layer uses batch normalization to improve the performance and stability of the neural network. The activation function uses the ReLU function [28]. To avoid the gradient disappearance, the function of the first two convolutional layers is mainly to extract features, and the last one is to reduce dimensionality, which suppresses overfitting. In the portion of the fully connected layer, the neural unit mainly uses the previously extracted features to predict RUL. The activation function used in the first fully connected layer is also the ReLU function, and the dropout technology [29,30] is applied to avoid overfitting. The last fully connected layer is used to predict the RUL value.

### 2.4. Smooth Calibration

Taking into account the influence of many factors, such as noise, the collected data has a fixed deviation. The neural network predicts life based on the collected data, so the predicted remaining life curve fluctuates up and down throughout the cycle. In practice, the RUL of the machine should be stable. Even if it encounters the interference of some factors, the life curve fluctuates in very few local places. Because of this phenomenon, we should consider that there should be a caching relationship between the RUL at the past moment and the RUL at the current moment. Inspired by the momentum gradient descent, the article proposes a momentum smoothing method for the RUL for the test set. The formula is as follows.

(8) $$predict_{t}=k\times y_{t}+(1-k)\times predict_{t-1},0\leq k\leq 1$$

where *y_t_* is the predicted value at time *t* by using dual-channel LSTM, *predict_t_*_−1_ is the predicted outcome after smoothing at the last time, *predict_t_* is the value after smoothing at the current time *t*, and *k* represents the proportion of *y_t_* in *predict_t_*. The larger the *k* is, the smaller the buffer of the previous RUL will be at the current moment; the opposite is true the smaller the value of *k* is.

### 2.5. Regularization

If the distribution of the acquired data deviates from the distribution of the actual data, the neural network model may be overfitting during training. This leads to the high accuracy of the training dataset but poor performance of the test dataset. In order to avoid this phenomenon, we can consider using regularization [31,32] methods.

The regularization methods include the dropout technology, the L2 regularization, and the early stopping [33] method of the partition verification set in this model. The dropout technology is to make the activation value of the neuron stop working with a certain probability during the forward propagation, which can make the model more generalized since it does not rely too much on some local features.

L2 regularization is also called weight attenuation, which makes the solution of the model biased towards weights with smaller norms, and limits model space by limiting the size of the weight norms, thereby avoiding overfitting to a certain extent. The formula is as follows.

(9) $$C=C_{0}+\frac{\lambda }{2n}\sum_{w}w^{2}$$

Among them, *C*_0_ represents the loss function of the model. In the proposed method, *C*_0_ is the MSE; w represents the weight parameter of the model; n represents the number of parameters; λ is the regular term coefficient; *C* represents the loss function after adding L2 regularization.

In the process of training the model, the loss function keeps getting smaller and the parameters keep approaching the optimal solution, but it is possible that at a certain gradient contour, the model has reached the optimal solution in a fixed spatial range. If we continue to train at this time, the model may linger near the optimal solution or even overfit. Considering this phenomenon, we can divide the training set into a training set and a validation set, and the division ratio is p1: p2. When the model is in p training cycles, and the loss value of the validation set has not decreased, the training is halted.

### 2.6. Dual-Channel LSTM Model Construction Process

The flow chart of the dual-channel LSTM model construction is described step by step below.

(1) Firstly, solve the prognosability of features and select useful features. Then the data are standardized by z-score, and the data are scaled to the same metric to speed up the training of the model. After that, set a reasonable RUL threshold to distinguish the health status of the equipment. Finally, divide the data into feature value Input_1_ = [*x*_1_, *x*_2_, …, *x_Nf_*] and feature difference window Input_2_ = [*d*_1_, *d*_2_, …, *d_Nf_*] to establish the input and output pair of the dual-channel LSTM model for model training and testing.
(2) Split the training set of each sub-dataset into a training set and a validation set. The training set is used for parameter training of the model, and the validation set is used to check whether the model converges. If it converges, stop training the model.
(3) Construct a dual-channel LSTM network model. LSTM executes parallel operations with difficulty, so training LSTM takes more time. In order to speed up the training of the model as much as possible, the number of LSTM layers is set to 1. At the same time, in order to assure that the LSTM effectively extracts the information of the sequence data, through the prediction accuracy of the model on the dataset, the dimension hidden_size of the hidden layer of the LSTM is adjusted.
(4) On the training set, apply the mini-batch gradient descent method and Adam algorithm to train the dual-channel LSTM network model. In order to avoid model overfitting, L2 regularization constraints are applied to the model parameters and dropout technology is added to the fully connected layer.
(5) Use the trained model to predict the RUL of the test set. This paper proposes a momentum-smoothing modular smooth neural network prediction result.
(6) Use the scoring function (score) and the root mean square error (RMSE) to evaluate the performance of the model. The evaluation index formula is as follows.

(10) $$\begin{array}{l} s=\;\sum_{i=1}^{N}s_{i},\;s_{i}\;=\;\left\{\begin{array}{c} e^{-\frac{d_{i}}{13}}-1,\;\mathrm{for}\;d_{i}\;<\;0\\ e^{\frac{d_{i}}{10}}-1,\;\mathrm{for}\;d_{i}\geq 0 \end{array}\right.\;\\ d_{i}=predict_{i}-RUL_{i}\\ RMSE=\sqrt{\frac{1}{N}\sum_{i=1}^{N}d_{i}^{2}} \end{array}$$

Among them, *predict_i_* represents the predicted value, *RUL_i_* represents the ground *RUL*, and *N* represents the number of all sample data.

**Figure 5:**
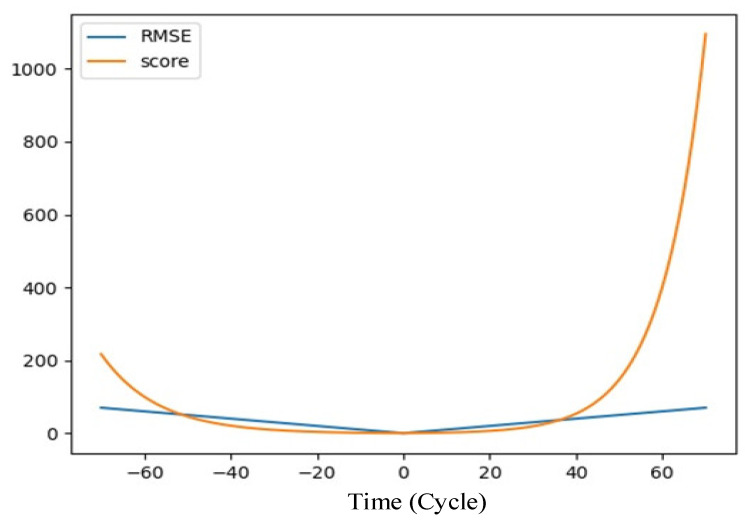
Evaluation index function.

Figure 5 visualizes the relationship between the evaluation index function. It can be seen that the score evaluation index and di are exponential. Compared with *RMSE*, the penalty for high deviation is raised and the penalty for low deviation is reduced in the score function.

**Figure 6:**
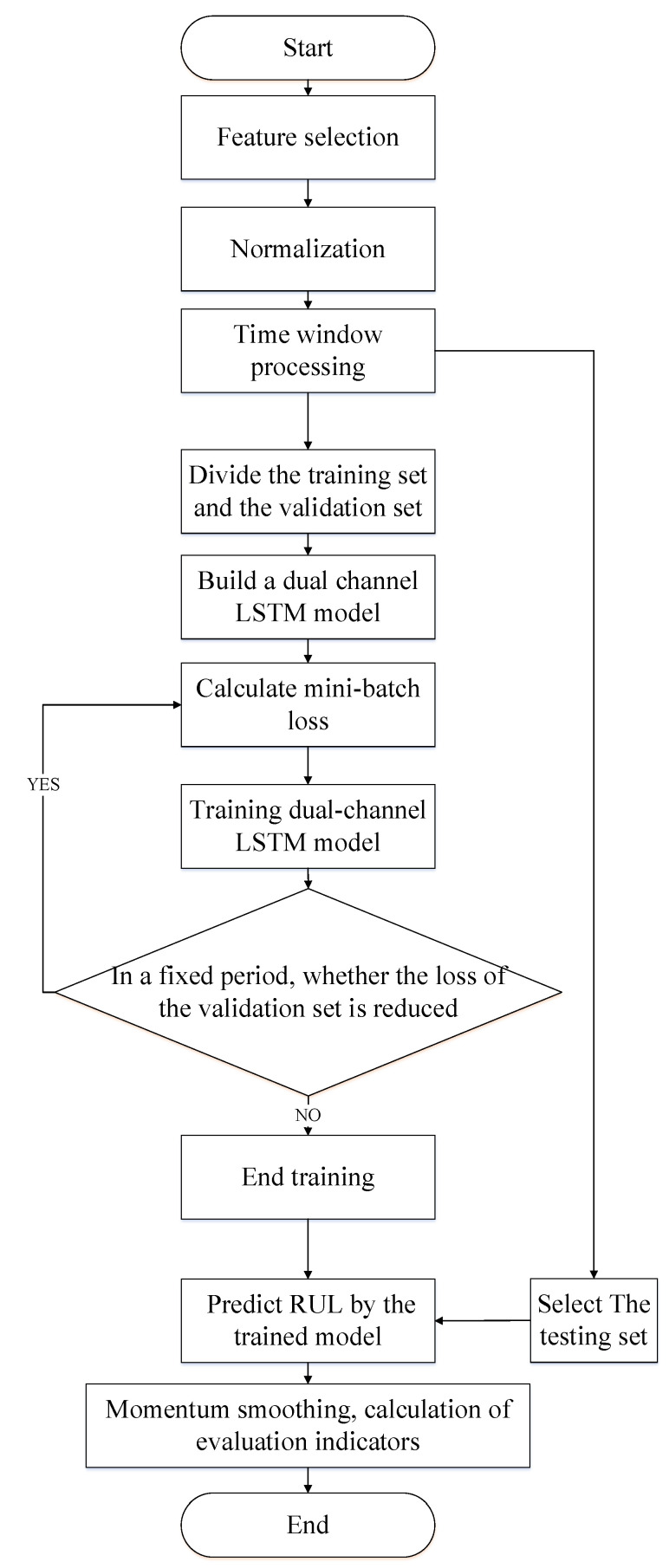
Flowchart of dual-channel LSTM.

LSTM perform parallel operations with difficulty, so training LSTM takes more time. To speed up the training of the model as much as possible, the number of LSTM layers is set to 1. At the same time, in order to ensure that the LSTM effectively extracts the information of the sequence data through the prediction accuracy of the model on the dataset, the dimension hidden size of the hidden layer of the LSTM is adjusted. Meanwhile, batch size and learning rate are adjusted according to the prediction accuracy of the model. Figure 6 depicts the flowchart of dual-channel LSTM.

## 3. Experiment and Result Analysis

In order to verify the effectiveness of dual-LSTM method, it is applied to predict the RUL of a turbine engine.

### 3.1. Description of C-MAPSS

**Figure 7:**
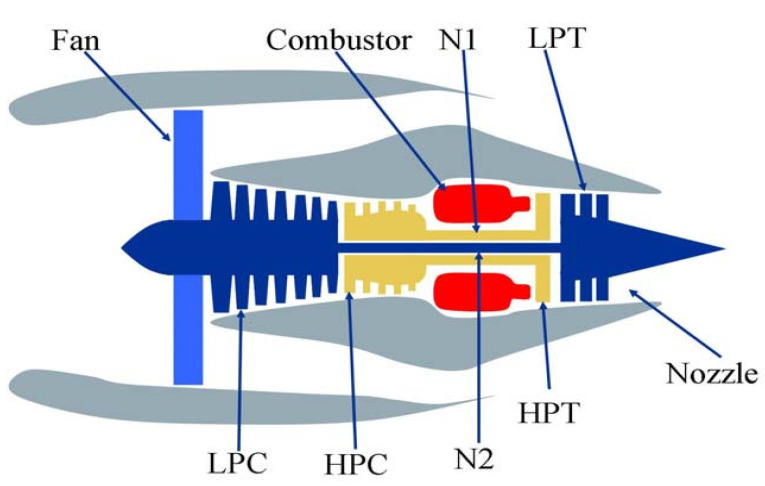
Simplified diagram of engine simulated in C-MAPSS.

C-MAPSS simulates a 90,000-pound thrust-type engine model [34]. The built-in control system includes a fan speed controller and a set of regulators and limiters. Figure 7 shows the main components of the engine model. Table 1 lists the 21 sensors that monitor engine conditions.

From Table 2, we can see that the C-MAPSS dataset is composed of four diverse sub-datasets: FD001, FD002, FD003, and FD004. The number of engine units in each sub-dataset is unique; the numbers of failure modes and operating settings are also distinct. Each sub-dataset is divided into a training dataset and a test dataset, recording the true RUL of the scroll engine from a healthy state to a degraded state at each moment. Each sub-dataset is divided into training datasets and test datasets, which at each moment record three operational settings and the data of the 21 sensors of the scroll engine unit. The FD001 and FD002 sub-datasets contain one failure mode (HPC degradation), while FD003 and FD004 contain two degradation modes (HPC degradation and Fan degradation). There is only one running condition for FD001 and FD003, and there are six running conditions for FD002 and FD004. Due to the complex and changeable operating environment of the FD002 and FD004 sub-dataset engine unit, it is more difficult to estimate the RUL of the FD002 and FD004 sub-datasets.

### 3.2. Life Prediction on C-MAPSS Datasets

Table 3 shows the detailed parameters of the proposed dual-channel LSTM. The length of the test set is determined by the size of the time window and the window moving step [12]. The parameters of the model are initialized by setting a random seed. Then, through training the model and adjusting the parameters, the parameters in Table 3 are finally determined. There are 16,324 parameters in the model, with a total size of 28.92 M.

The experiment is processed on a PC with Intel Core i5-5200U CPU, 12 GB RAM, and NVIDIA GeForce 910M GPU (on the PyTorch 1.2.0).

After training the model, we take the FD001 sub-dataset as an example to test the effect of the method. The following shows the RUL curve of the engine units 24, 34, 76, and 100 in the FD001 test set.

**Figure 8:**
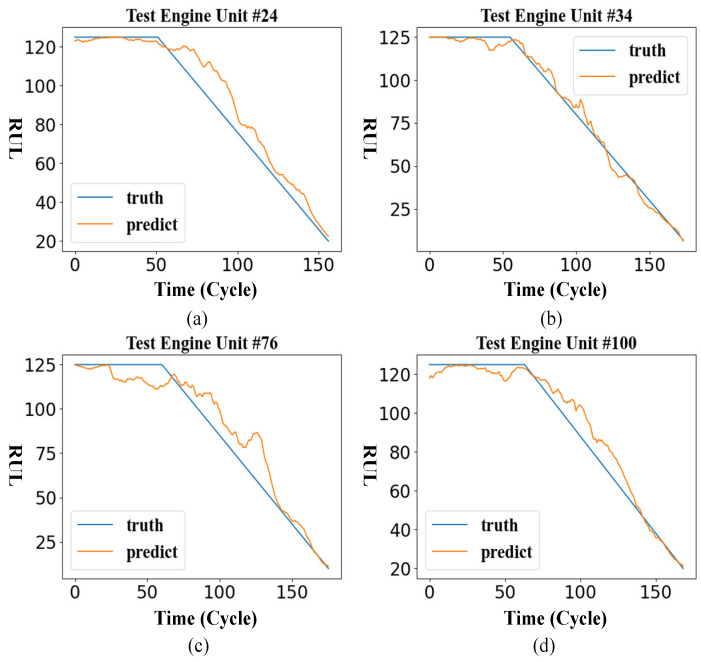
The remaining service life curve of some FD001 engines. (**a**) Engine Unit 24 RUL prediction curve; (**b**) Engine Unit 34 RUL prediction curve; (**c**) Engine Unit 76 RUL prediction curve; (**d**) Engine Unit 100 RUL prediction curve.

It can be seen from Figure 8 that the results predicted by the model are constant in the early stage. In the later stage, the predicted outcome shows a linear decline, which is in line with the overall trend of the ground RUL curve.

### 3.3. Compared with State-of-the-Art Methods

In this research part, we use different advanced research means to compare the dual-channel LSTM method proposed in the article.

As shown in Table 4, our proposed dual-channel LSTM method outperforms most methods in terms of RMSE and score. Compared with other methods, the dual-channel LSTM method adds a time feature difference input channel, which can extract key information from the time feature speed to reduce the error in RUL prediction. In the sub-datasets FD002 and FD004 with multiple faults and complex operating settings, the dual-channel LSTM method reduces the RMSE values to 17.63 and 17.41, respectively, and the scores to 1773.47 and 2617.45, respectively. This reflects that when the environment becomes bad, the proposed dual-channel LSTM method still performs well. The main reason is that the dual-channel recurrent neural network not only considers the time value of the time feature but also thinks about the difference of the time feature at different times, which reduces the impact of the environment on the model. Generally speaking, the dual-channel LSTM method has certain advantages compared to the other methods in the table.

### 3.4. Model Analysis

In this section, we use the test set of the sub-dataset FD001 as an example to analyze the model.

#### 3.4.1. Mini-Batch Size

In the training process, the training set is divided into small batch samples for training. Table 5 shows the effect of batch processing on the prediction results of the model.

**Figure 9:**
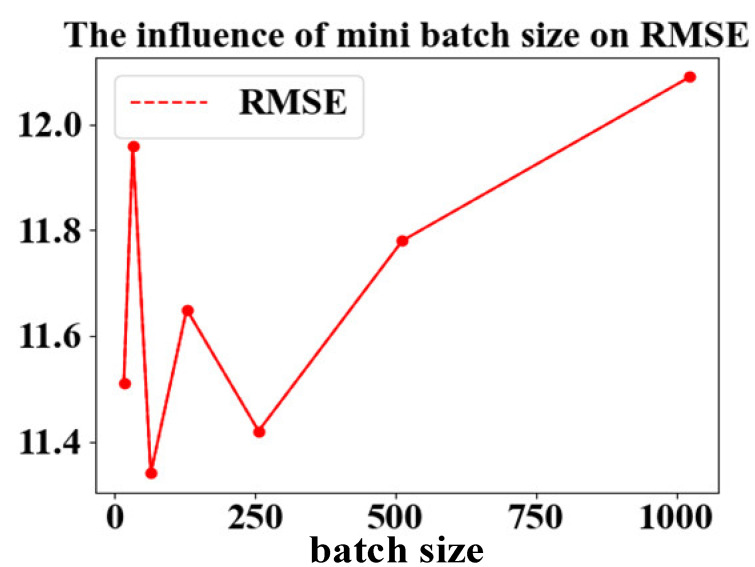
The effect of mini-batch size.

In Table 5, when mini-batch equals 64, the RMSE and score of FD001’s test set are the lowest. Therefore, the value of mini-batch in this method is 64. The results are depicted in Figure 9.

#### 3.4.2. Momentum Smoothing

This experiment studies the influence of momentum smoothing on the predicted results under the same network structure. Table 6 describes the results of the experiment.

**Figure 10:**
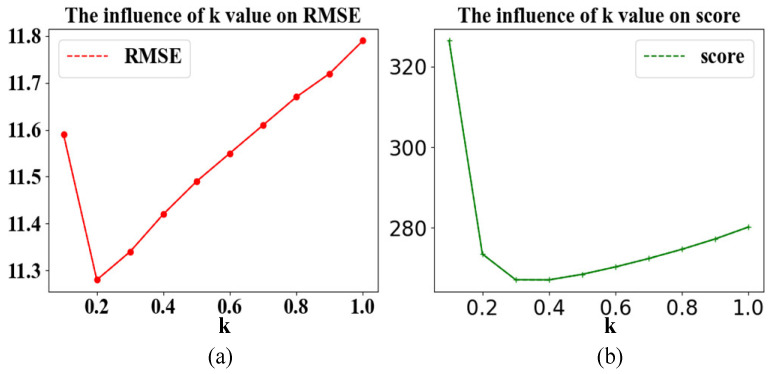
The effect of the k value. (**a**) The influence of k value on RMSE; (**b**) the influence of k value on score.

When k = 0.3, the RMSE of the test set of FD001 dropped from 11.79 to 11.34, and the score dropped from 280.2 to 267.1. It shows that the use of momentum smoothing can predict the RUL more accurately. At the same time, as k decreases, RMSE and score first decrease and then increase. This shows that when k is greater than or equal to 0.5, reducing the value of k (that is, increasing the buffering effect of the RUL of the previous period on the current) can lower the RMSE and score. However, when the value of k is small, continuing to reduce the value of k makes the proportion of the current RUL predicted by the model too low, resulting in the RUL being determined by the previous RUL after smoothing. So, the score rose sharply. The results are visualized in Figure 10.

**Figure 11:**
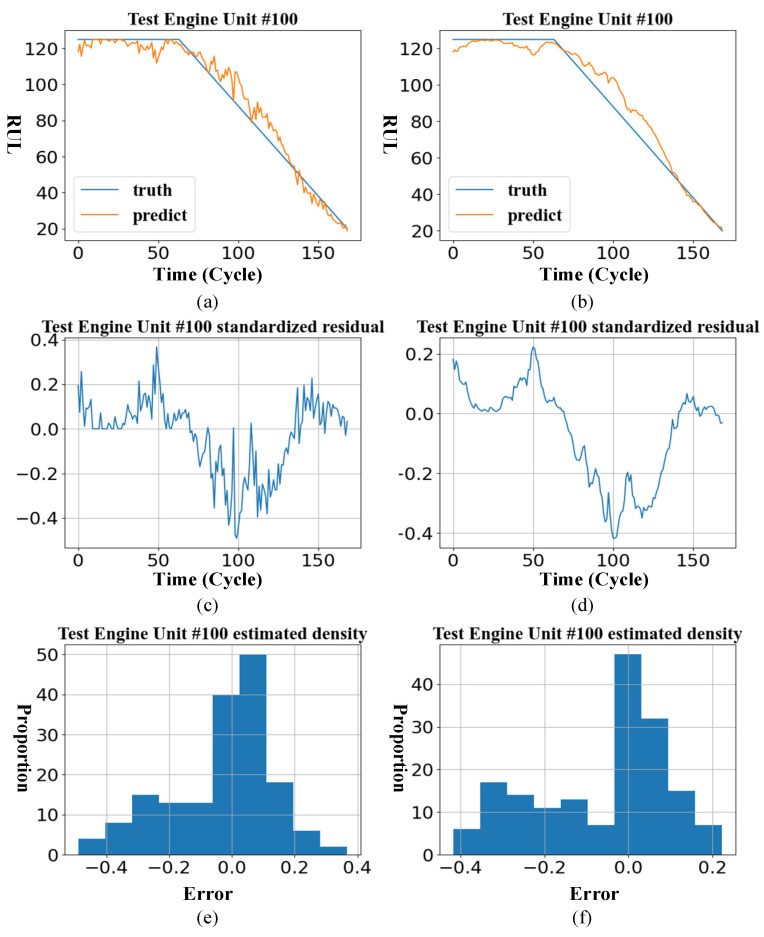
Effect before and after momentum smoothing. (**a**) RUL curve before smoothing; (**b**) RUL curve after smoothing; (**c**) model standardized residual before smoothing; (**d**) model standardized residual after smoothing; (**e**) histogram of the standardized residual before smoothing; (**f**) histogram of the standardized residual after smoothing.

In order to see the effect of momentum smoothing, take the engine unit 100 of the test set in the sub-dataset FD001 as an example to visualize the results after using momentum smoothing in Figure 11.

Due to the addition of the momentum-smoothing module in the proposed method, the historically predicted RUL affects the current predicted RUL, which reduces the randomness of the predicted results, so the residual curve is relatively smooth.

#### 3.4.3. Stop Early

**Figure 12:**
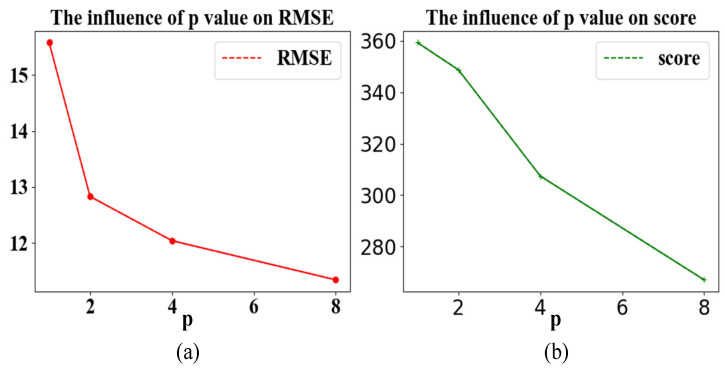
The effect of the *p* value. (**a**) The influence of *p* value on RMSE; (**b**) the influence of *p* value on score.

In order to verify that early stopping is beneficial for preventing the model from overfitting, we are now conducting research to set p to take a different value within 180 epochs; if the loss of the verification set does not decrease in p cycles, stop training and use the test set to check the effect. Letting p take None is equivalent to letting the model train for 180 epochs; that is, the early stopping method is not used. The results are visualized in Figure 12.

It can be summarized from Table 7 that as the *p*-value increases, the evaluation indicators RMSE and score are both decreasing. This is due to the use of the mini-batch gradient descent rule, which has randomness; the verification set may not drop continuously within a fixed training period. Therefore, the *p*-value needs to be enlarged to reduce randomness. However, it can be found that when the *p*-value increases to a definite value, the RMSE and score does not decrease significantly as the *p*-value continues to become larger because the randomness has been reduced to a negligible level. If model performance is similar, the training time should be as short as possible, and p should be set to a reasonable value, i.e., not the larger the better. The effect of the model trained in 180 epochs is not much different from the one of using the early stopping strategy, indicating that the model has reached a fixed spatial optimal solution before 180 epochs, but with the early stopping strategy, the model normally stops training before 80 epochs, which saves more than half of the training time.

#### 3.4.4. Optimizer

**Figure 13:**
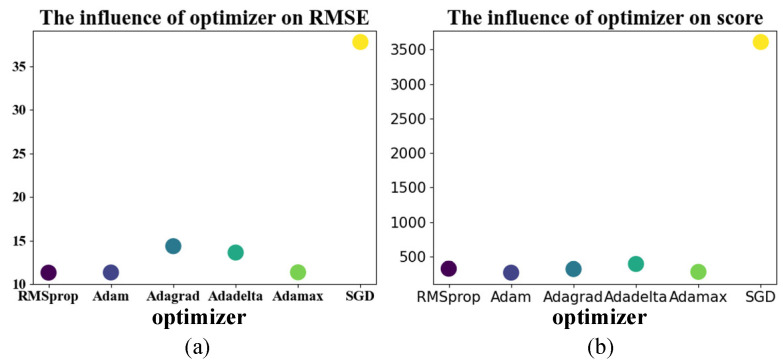
Effect of optimizer. (**a**) The influence of optimizer on RMSE; (**b**) the influence of optimizer on score.

It can be seen from Table 8 that the use of the SGD optimizer converges to a locally optimal solution. RMSE only dropped to 37.5, while the RMSE can be dropped to less than 15 using several other optimizers. Using the RMSprop optimizer, the RMSE can be reduced to 11.32, but the score is higher than that using the Adam optimizer, so the Adam optimizer is finally used for the gradient descent of the method. The results are visualized in Figure 13.

#### 3.4.5. Model Generalization

In order to verify the generalization of the model, take the logarithm of RUL, let $\mathrm{RUL}=\ln\left(\mathrm{RUL}+1\right)$, and train the model again. The results are as follows.

**Figure 14:**
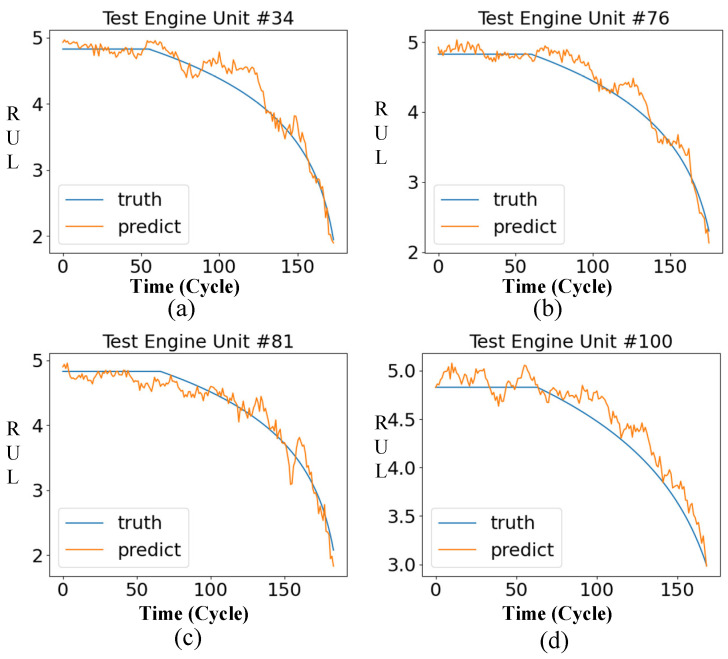
The remaining service life curve of some FD001 engines. (**a**) Engine Unit 34 RUL prediction curve; (**b**) Engine Unit 76 RUL prediction curve; (**c**) Engine Unit 81 RUL prediction curve; (**d**) Engine Unit 100 RUL prediction curve.

In Table 9, after taking logarithms of RUL, the RMSE value of the test set of the sub dataset FD001 is 0.15 and the score value is 1.3. It can be seen from Figure 14 that the model still fits the new RUL.

## 4. Discussion and Summary

This paper proposes a new network model dual-channel LSTM. In this neural network, dual-channel LSTM is used to deal with the time feature and its first-order feature and solve the problems of gradient disappearance and gradient explosion during long sequence training; CNN considers the relationship in the time dimension and integrates the features of multiple time moments into one dimension, which plays a role in dimension reduction. Aiming at the jagged phenomenon of the RUL curve predicted by the neural network, a momentum-smoothing method is proposed to deal with RUL curves and improve the accuracy of prediction. The dual-channel LSTM improves the learning ability of network because it learns the information of the two dimensions of the time features, which has good application scenarios in actual industrial environments. However, there are still some questions that need to be resolved. Firstly, in momentum smoothing, the ratio k is adjusted according to the effect. Whether there is a precise method to determine the value of k or not is not known. Secondly, the application of deep learning to industry requires a large amount of data. Then, the acquisition of industrial data is complicated. Therefore, how to apply deep learning with a small amount of data is also a current problem. Thirdly, there are many types of industrial data. Using the same model to process different types of data, the effect may not be ideal. The difficult point is whether it is possible to propose a data fusion method to improve the universality of the model, but this problem will be explored in future research.

## Figures and Tables

**Table 1:** Sensor introduction.

Index	Description	Symbol
1	Total temperature at fan inlet	˚R
2	Total temperature at LPC outlet	˚R
3	Total temperature at HPC outlet	˚R
4	Total temperature at LPT outlet	˚R
5	Pressure at fan inlet	psia
6	Total pressure in bypass-duct	psia
7	Total pressure at HPC outlet	psia
8	Physical fan speed	rpm
9	Physical core speed	rpm
10	Engine pressure ratio (P50/P2)	-
11	Static pressure at HPC outlet	psia
12	Ratio of fuel flow to Ps30	pps/psi
13	Corrected fan speed	rpm
14	Corrected core speed	rpm
15	Bypass ratio	-
16	Burner fuel–air ratio	-
17	Bleed enthalpy	-
18	Demanded fan speed	rpm
19	Demanded corrected fan speed	rpm
20	HPT coolant bleed	lbm/s
21	LPT coolant bleed	lbm/s

This dataset is simulated using the commercial modular aerospace propulsion system simulation. Details about the dataset are as follows.

**Table 2:** Dataset introduction.

Sub-Datasets	FD001	FD002	FD003	FD004
Engine units in the training dataset	100	260	100	249
Engine units in the test dataset	100	259	100	248
Fault modes	One (HPC Degradation)	One (HPC Degradation)	Two (HPC Degradation, Fan Degradation)	Two (HPC Degradation, Fan Degradation)
Conditions	One (Sea Level)	Six	One (Sea Level)	Six
Training samples	17,731	48,819	21,820	57,522
Test samples	100	259	100	248

**Table 3:** Parameter details.

Parameter	Value	Parameter	Value
Nt for FD001 to FD004	30/20/30/15	Batch size	64
Nf for FD001 to FD004	17/24/18/24	Learning rate	0.001
hidden_size	32	RUL threshold	125
k for FD001 to FD004	0.3/0.5/0.4/0.5	p	8
L2 normalization	0.0001	Activate function	ReLU

**Table 4:** Comparison of method effects.

Method	FD001		FD002		FD003		FD004	
	RMSE	Score	RMSE	Score	RMSE	Score	RMSE	Score
Dual-LSTM	11.34	267.1	17.63	1773.47	10.8	334.86	17.41	2617.45
Ensemble ResCNN [10]	12.16	212.48	20.85	2087.77	12.01	180.76	24.97	3400.44
AdaBN-DCNN [11]	13.17	279	20.87	2020	14.97	817	24.57	3690
MS-DCNN [12]	11.44	196.22	19.35	3747	11.67	241.89	22.22	4844
MA-LSTM [15]	13.52	258	22.57	3740	12.98	256	23.88	4560
Deep LSTM [15]	16.14	338	24.49	4450	16.18	852	28.17	5550
BiLSTM [15]	13.65	295	23.18	4130	13.74	317	24.86	5430
L(12.10.7.2)N(2) [35]	14.08	308	18.59	1880	12.15	221	20.91	2633
Semi-supervised [36]	12.56	231	22.73	3366	12.1	251	22.66	2840
DCNN [37]	12.61	273.7	22.36	10,412	12.64	284.1	23.31	12,466
MODBNE [38]	15.04	334.23	25.05	5585.34	12.51	421.91	28.66	6557.62
DBN [39]	15.21	417.59	27.12	9031.64	14.71	442.43	29.88	7954.51
LSTM [38]	16.14	338	24.49	4450	16.18	852	28.17	5550
MLP [39]	16.78	560.59	28.78	14,026.72	18.47	479.85	30.96	10,444.35
Growing RNN [40]	14.31	302.8	23.71	4105.5	16.42	457.49	27.95	4906.04
VarSeq LSTM [40]	15.23	250	22.87	4532	14.53	1523	26.11	5627

**Table 5:** Performance of mini-batch size.

**Mini-Batch**	16	32	64	128	256	512	1024
**RMSE**	11.51	11.96	11.34	11.65	11.42	11.78	12.09
**Score**	283.22	323.53	267.1	297.3	286.4	319.46	344.24

**Table 6:** The effect of the k value.

**k**	0.1	0.2	0.3	0.4	0.5	0.6	0.7	0.8	0.9	1
**RMSE**	11.59	11.28	11.34	11.42	11.49	11.55	11.61	11.67	11.72	11.79
**Score**	326.44	273.48	267.1	267.07	268.45	270.29	272.37	274.67	277.24	280.2

**Table 7:** Performance of different *p*-values.

***p***	1	2	4	8	None
**RMSE**	15.58	12.83	12.04	11.34	12.17
**Score**	359.26	348.66	307.39	267.1	343.03

**Table 8:** Performance of different optimizers.

	RMSprop	Adam	Adagrad	Adadelta	Adamax	SGD
**RMSE**	11.32	11.34	14.36	13.63	11.36	37.8
**Score**	324.26	267.1	318.92	392.83	278.51	3607.59

**Table 9:** Performance of different RULs.

RUL	RUL	ln(RUL + 1)
**RMSE**	15.58	0.15
**Score**	359.26	1.3

## Data Availability

Not applicable.
